# Does Urodynamic Stress Incontinence Increase After the Menopause?: Results from 2,994 Urodynamic Studies in Australian Women

**DOI:** 10.1007/s00192-024-05876-3

**Published:** 2024-07-23

**Authors:** Nevine te West, Katie Harris, Michael Chapman, Kate Hilda Moore

**Affiliations:** 1 St George Hospital, School of Women’s and Children’s Health, University of New South Wales, Sydney, Kogarah Australia; 2 The George Institute for Global Health, University of New South Wales, Sydney, Australia

**Keywords:** Prevalence, Urodynamics, Incontinence, Pre-menopause, Post-menopause

## Abstract

**Introduction and Hypothesis:**

Most studies attempting to estimate the age-related prevalence of urinary incontinence (UI) have used questionnaires. In the present study we analysed a consecutive series of urodynamic test results to determine the distribution of the different types of UI in pre- and post-menopausal women. We hypothesised that the prevalence of urodynamic stress incontinence (USI) would be significantly greater in pre-menopausal than in post-menopausal women.

**Methods:**

All women from a large tertiary urogynaecology department, who underwent urodynamic tests during the years 2000–2015 were included. Patient history and test results were collected. A sample size of 1,475 was calculated, based on the hypothesis that the prevalence of USI will be 20% larger in the pre- versus the post-menopausal group.

**Results:**

A total of 2,994 women with UI on urodynamics were available. There was a significant difference between pre- and post-menopausal status for each of the three diagnoses: USI 483 (59.3%) versus 912 (41.8%), detrusor overactivity (DO) 125 (15.4%) versus 399 (18.3%) and USI with concomitant DO 206 (25.3%) versus 869 (39.9%). A bimodal pattern of age was seen in women with USI, with a peak in the 46–50 and 61–65 age group, before decreasing with age. DO generally increased with age. USI with concomitant DO increased steadily after the menopause, becoming the predominant type after the age of 66.

**Conclusions:**

In this large cohort of women attending urodynamics, we have shown that USI is the predominant type of incontinence in pre-menopausal women; however, USI with concomitant DO increases after menopause, eventually predominating.

## Introduction

Urinary incontinence (UI) is a common, debilitating condition affecting the quality of life of about a third of women in Australia across all ages [1]. With the ageing of the population, incontinence is becoming an increasing problem. In Australia, almost 2 out of 5 women aged 65 and over complain of severe incontinence, compared with 2% in 40- to 64-year-olds [2]. Many governments subsidise incontinence pads through continence aids schemes; for example, $83.5 million was spent in Australia between 2014 and 2015 [3]. Therefore, the age-related prevalence of UI is receiving increased international interest.

The vast majority of studies attempting to estimate the age-related prevalence of UI predominantly in women have used self-reported questionnaires to determine the degree or type of incontinence. The EPIC study of more than 19,000 participants across five countries (Canada, Germany, Italy, Sweden, UK; *N* = 19,165) using telephone interviews based on International Continence Society (ICS) terminology found stress urinary incontinence (SUI) to be the most common type in the total population (6.4%) followed by mixed urinary incontinence (MUI; 2.4%) and then urge urinary incontinence (UUI; 1.5%) [4]. SUI was also the most prevalent in all age groups surveyed: 3.7% < 39 years, 7.9% 40–59 years and 8% > 60 years. These findings agreed with a common perception that incontinence is more common after the menopause: “70% of women relate the onset of incontinence to their final menstrual period” [5].

However, estimates from other studies varied, possibly because of the different definitions used for the types of incontinence, the countries studied and the variations in the methods of survey. Self-reported questionnaire information on UI is known to have several limitations. First, it reflects symptoms, rather than a diagnosis, for which urodynamic studies are essential [6]. Second, self-reporting may encourage under-reporting of lower urinary tract symptoms owing to embarrassment and unwillingness to report UI [7]. Last, the mode of questionnaire administration may also affect the quality of the study [8].

In contrast, two publications regarding age-related prevalence of UI have utilised urodynamic diagnoses [9, 10]. However, these publications appeared unusual, with a 20% rate of voiding dysfunction, which is a high rate for the female population.

As part of the growing awareness of age trends in incontinence, there has been increasing interest in the use of vaginal oestrogen in post-menopausal women [11–14], which has benefit as a first-line treatment for incontinence [15]. Previous authors have mentioned difficulty in recruiting women with stress incontinence after the menopause into studies [14]. Theoretically, oestrogen deprivation that occurs after the menopause should be associated with reduced epithelial coaptation of the urethral walls, reduced vascular pressure in the urethra and weakening of the ligaments supporting the peri-urethral tissues [16]. Thus, we have been interested in assessing urodynamic stress incontinence (USI) after the menopause. However, most of the previous studies employing questionnaires or urodynamics have not focussed on the menopause as a possible contributing factor to the prevalence of any type of incontinence.

The aim of the present study was to analyse a consecutive series of urodynamic test results to determine the distribution of the different types of UI in pre- and post-menopausal women, more precisely than previous studies.

We hypothesised that the prevalence of USI in this population would be significantly greater in pre-menopausal women than in post-menopausal women.

## Materials and Methods

All women from a large tertiary urogynaecology department in Australia, who underwent video-urodynamics or cystometry during the years 2000–2015, because of UI, were included in the study. The department does not accept referrals for neuropathic incontinence, such as spinal cord injuries or multiple sclerosis. The study was approved by the local health district research ethics committee (reference LNR 2020/ETH00044). In this academic urogynaecology department, all patient histories were recorded on a set proforma; these records were stored in the department. All urodynamics studies were performed as per the ICS guidelines [17] in a standardised manner and all practitioners had been trained by the same urogynaecologist during the 15 years of data collection. Dipstick urine analysis was performed prior to the study and a midstream urine sent for analysis. If the dipstick suggested a urinary tract infection, urodynamics was postponed and antibiotics given.

The urodynamics study results were also reported in a standardised proforma. The reports were reviewed by the authors and the data collated and entered into a database in SPSS Statistics 26. The corresponding patient history proformas were used to obtain data on patient demographics, presenting symptoms, duration of symptoms, menstrual status at first visit, previous continence and prolapse procedures, current hormone replacement therapy (HRT) and topical oestrogen use. As per departmental protocol and good practice guidance [18], all women who had not previously trialled conservative management were referred for pelvic floor physiotherapy/bladder training at their first visit. Post-menopausal women not using topical oestrogen were commenced on oestriol cream, unless contraindicated. It was presumed that these patients were still using oestriol cream when their study was performed. Of the women who had undergone multiple urodynamics studies in our department, the first study was included in these analyses. Women without UI were excluded, i.e. patients with voiding dysfunction or undergoing urodynamics prior to prolapse surgery for de novo SUI. Therefore, there were three possible diagnoses at urodynamics: USI, detrusor overactivity (DO) and USI + DO.

Women who were still menstruating were recorded as pre-menopausal. If women were no longer menstruating owing to a hysterectomy with concomitant bilateral salpingo-oophorectomy (BSO) they were considered post-menopausal. In Australia the average age at menopause is 51 years [19, 20]. Women below the age of 51 who were not menstruating owing to a previous hysterectomy were noted as pre-menopausal if they had not had a BSO. Women aged ≥ 51 years who had undergone a hysterectomy without BSO were considered menopausal. When known, age at menopause was used (no menstruation for 12 months).

Because our tertiary unit receives referrals from a 400-km radius and because many of the patients from distant locations are more complex (e.g. less likely to have simple stress incontinence), analysis by secondary versus tertiary referral was performed. It was hypothesised that tertiary referrals from a much broader area might involve patients with more complex incontinence (i.e. mixed incontinence) compared than more local secondary referrals. Within this subset analyses, local referrals were selected by postal code from within the department’s catchment area compared with the tertiary referrals from outside the catchment area.

### Statistical Methods

The different types of incontinence (USI, DO, USI + DO) prevalent in these women were calculated as the number of women experiencing the respective types of incontinence divided by the number of incontinent women in this study multiplied by 100 and presented as a percentage.

Patient characteristics and demographics of the women were assessed. Data were summarised by types of incontinence, continuous data are presented as mean (SD) or median (IQR), for normally distributed and skewed data respectively. Categorical variables are presented as number and percentage. The distribution of age grouped in 5-year intervals was plotted as a histogram for each type of incontinence separately.

The percentage of each type of incontinence was calculated by the following sub-groups: by pre- versus post-menopause, by age group (i.e. 40–45, 45–50, 50–55, 56–60) and by secondary versus tertiary referral. The difference in percentage of incontinence types between subgroups was analysed using Chi-squared tests.

Additionally, as a sensitivity analysis we assessed the proportions of the three types of UI using the 51 years cut-off as a proxy for menopause status and repeated the calculation excluding all women with unclear data regarding their menopausal status. (For the outcomes, see Tables 3 and 4 in Appendix.

The risk factors for different types of incontinence were modelled simultaneously in a multinomial logistic regression model. Multinomial logistic regression models were selected because we have a nominal (unordered categorical variable) outcome variable (UI) with three categories USI, DO and USI + DO. The log odds of the outcomes are modelled as a linear combination of predictor variables. The baseline outcome (comparator) category was selected as USI + DO. Three sets of models were fit:

1. Univariate (overall and within the pre- and post-menopausal groups) in order to ascertain the risk of type of incontinence overall and by menopausal status.
2. Adjusted for menopausal status—pre- and post-menopause—to ascertain the effect of menopause on incontinence type.
3. Additionally adjusted for parity, body mass index (BMI), a history of hysterectomy, HRT, topical oestrogen use, chronic cough and constipation to determine if the effect of menopausal status changed after adjustment for confounders.

Results from these models are presented as odds ratios (ORs) with 95% confidence intervals (CIs).

### Sample Size

We hypothesised that the prevalence of USI in this population will be 20% higher in the pre-menopausal group versus the post-menopausal group (i.e. 60% versus 40%), for which a sample size of 1,475 would be required to detect a statistically significant difference between the two proportions.

## Results

A total of 3,546 consecutive women underwent urodynamics between 2000 and 2015 (all records were obtained). Of these, 546 (15.4%) were diagnosed without UI but with the following diagnoses: voiding dysfunction (*n* = 128, 3.6% of the total), sensory urgency (*n* = 197, 5.6%) and normal urodynamics studies (*n* = 221, 6%) were excluded. Additionally, 6 women with a main complaint of urgency frequency prior to the test were excluded. This left 2,994 women who had incontinence, 1,395 (47%) had USI, 524 (17%) DO and 1,075 (36%) USI + DO (Table 1). Patient demographics are also summarised in the table. Women with USI were on average younger (57.9 years) than those with DO (60.2 years) and USI + DO (64.6 years). The women were predominantly white and 330 (11%) had undergone previous continence surgery. The rate of chronic cough was higher in those with USI and USI + DO, and constipation was higher in those with DO. Note that 12.1% of incontinent women were using systemic HRT, with a significant difference between the urodynamic groups (*p* < 0.001). The vast majority of urodynamic tests were performed within 3 months of history taking.

**Table 1:** Population demographics

Characteristics	All women, *N* = 3,546	Women with incontinence, *n* = 2,994	Women with USI, (*n* = 1,395	Women with DO, *n* = 524	Women with USI + DO, *n* = 1,075
Age in years, mean (SD)	59.9.0 (14.6)	60.5 (14.6)	57.9 (13.9)	60.2 (15.7)	64.6 (13.9)
Parity, mean (SD)	2.5 (1.4)	2.5 (1.4)	2.6 (1.3)	2.2 (1.4)	2.6 (1.5)
BMI, mean (SD)	29.1 (15.6)	29.4 (16.8)	28.8 (9.3)	28.3 (6.8)	29.8 (6.5)
Age at menopause, mean (SD)	48.6 (5.7)	48.4 (5.9)	48.2 (5.3)	49.1 (5.4)	48.4 (6.2)
Prolapse, *n* (%)	972 (27.4)	788 (26.3)	368 (26.4)	125 (23.9)	295 (27.4)
Chronic cough, *n* (%)	411 (11.6)	354 (11.8)	166 (11.9)	40 (7.6)	148 (13.8)
Constipation, *n* (%)	963 (27.1)	806 (26.9)	382 (27.4)	160 (30.5)	264 (24.6)
On hormone replacement therapy, *n* (%)	430 (12.1)	364 (12.1)	205 (14.7)	46 (8.8)	113 (10.5)
Topical oestrogen at urodynamics, *n* (%)	1069 (30.1)	900 (30.1)	438 (31.4)	132 (25.2)	330 (30.7)

*USI* urodynamic stress incontinence, *DO* detrusor overactivity

Of the 2,994 women with incontinence, 2,112 (70.5%) lived locally to the study centre and 882 (29.5%) lived out of area. The proportions of the different types of incontinence in both local and non-local women were approximately the same: USI 979 (46.3%) versus 416 (47.1%), DO 377 (17.9%) versus 147 (16.7%), USI + DO 756 (35.8%) versus 319 (36.2%) respectively (Chi-squared *p* = 0.74). Therefore, these data were combined for all subsequent analyses.

As regards menopause status, of the 2,994 women 2,401 (80.1%) had a precise menopause status (e.g. 748 were pre-menopausal and 1,647 were post-menopausal). Unfortunately, there were unclear data on 599 (20.0%) owing to variability in data recording and due to some women having difficult recall about the onset of hot flushes or uncertainty regarding whether or not they were fully menstruating. Approximately half (*n* = 327) had undergone a hysterectomy without BSO. Of these 38 out of 327 (11.6%) were aged 32–50 and presumed pre-menopausal; 289 out of 327 (88.4%) were aged ≥ 51 and considered post-menopausal. The remainder (*n* = 272) had not had a hysterectomy or BSO. Of these 29 out of 272 (10.7%) were aged < 51 years and noted to be pre-menopausal and 243 out of 272 (89.3%) were aged ≥ 51 years and assumed to be post-menopausal.

The percentage of the three types of UI in relation to the determined pre- or post-menopausal status are presented in Table 2. The Chi-squared analysis shows that there was a statistically significant difference between pre- and post-menopausal status for each of the three diagnoses. Importantly, USI occurred in 59% of pre-menopausal women but only 42% of post-menopausal women (*p* < 0.001).

**Table 2:** Type of urinary incontinence using pre- and post-menopause where available and using 51 years as the menopause cut-off when unknown

Type of UI	Pre-menopause	Post-menopause	Total	*p* value (Chi-squared)
USI	483 (59.3)	912 (41.8)	1,395 (46.6)	< 0.001
DO	125 (15.4)	399 (18.3)	524 (17.5)	< 0.001
USI + DO	206 (25.3)	869 (39.9)	1,075 (35.9)	< 0.001
Total	814	2,180	2,994	< 0.001

Data shown are *n* (%)

Table 3 in Appendix shows similar data by menopausal age “cut-off” only (51 years)

*UI* urinary incontinence, *USI* urodynamic stress incontinence, *DO* detrusor overactivity, *MUI* mixed urinary incontinence

**Fig. 1:**
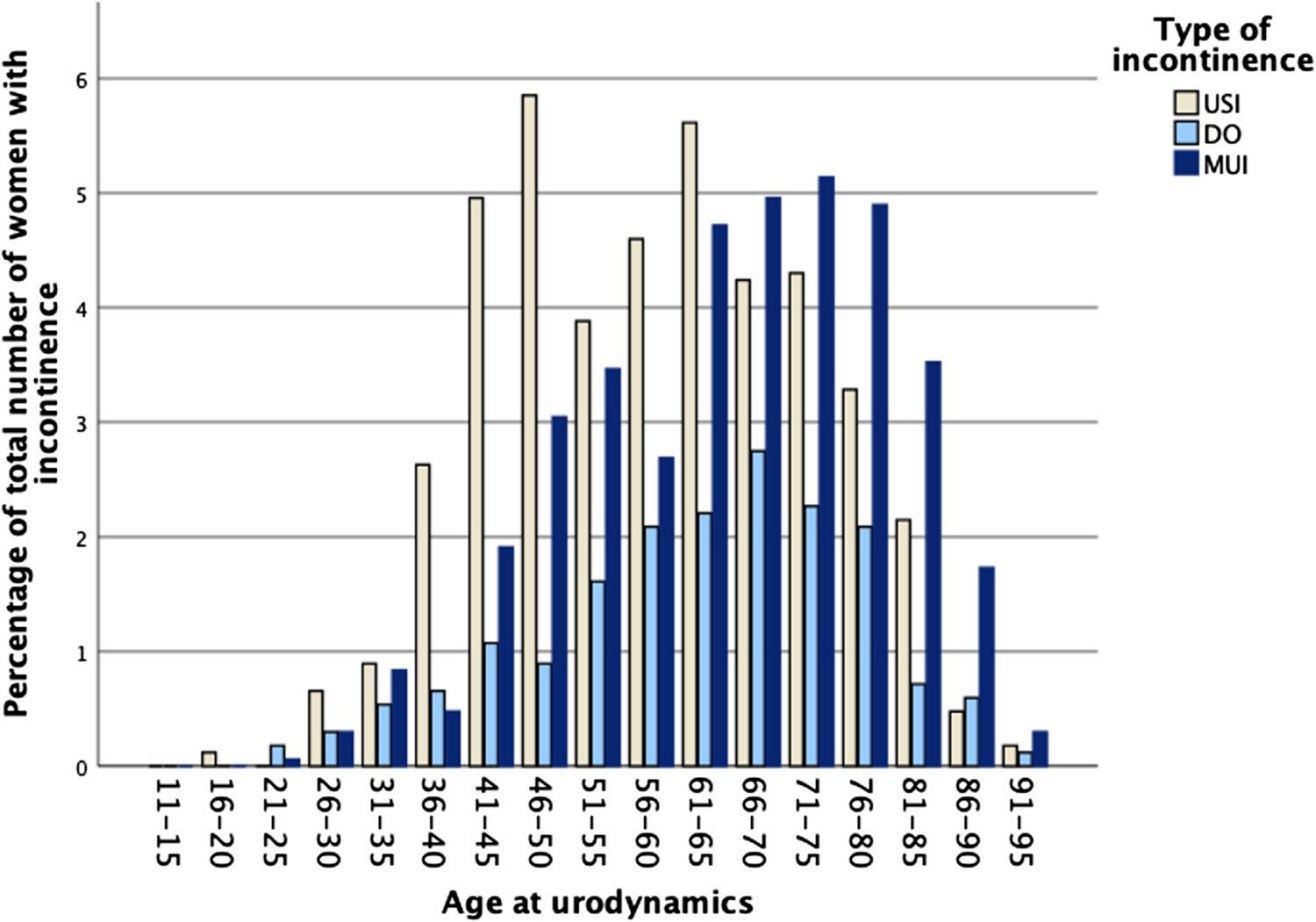
Histogram of the three types of incontinence per 5-year age groups. *USI* urodynamic stress incontinence, *DO* detrusor overactivity, *MUI* mixed urinary incontinence

Figure 1 shows a histogram of the age distribution, with the ages grouped per 5-year intervals by the three types of incontinence. The prevalence of USI peaked in the 46–50 age group, but fell in the post-menopausal groups (51–55 and 56–60), before increasing again in the 61–65 age group. In contrast, DO gradually increased with age, peaking in the 66–70 age group, before declining again. USI + DO eventually became the most prominent type of incontinence after the age of 66.

Using univariate multinomial logistic regression analysis with USI + DO as the baseline comparator, when stratified by menopausal status in pre-menopausal women, the odds of USI was double that of USI + DO (OR 2.34; 95% CI 1.99, 2.76). In contrast, when comparing DO with USI + DO, DO was 40% lower (OR 0.62 95% CI (0.50, 0.78)). However, when moving to post-menopausal women, the increase in USI disappeared (OR 1.05; 95% CI 0.96, 1.15). The OR (95% CI) of DO versus USI + DO was 0.46 (0.41, 0.52). These results correspond with the crude proportions of the three types of incontinence by menopausal status (Table 2).

Using multinomial logistic regression adjusted for menopause status only, the risk of USI (when comparing post-menopausal with pre-menopausal women) in relation to USI + DO was OR (95% CI) 0.45 (0.37, 0.54). When adjusted for the additional seven covariates (parity, BMI, history of hysterectomy, HRT, topical oestrogen, chronic cough and constipation), this finding still held OR (95% CI) 0.45 (0.34, 0.60).

As expected, the OR (95% CI) of DO (compared with USI + DO) was also lower in post-menopausal women than in pre-menopausal women, 0.73 (0.57, 0.93). As shown in Fig. 1 USI + DO rises even more than DO alone with age. However, after adjustment for these seven covariates this finding no longer held, OR (95% CI) 1.01 (0.70,1.47). In the multiple adjusted models (for DO vs USI + DO) there was a lower odds of DO for parous women than for nulliparous women, OR (95% CI) 0.84 (0.76, 0.94).

## Discussion

To our knowledge this is the largest study investigating urodynamics-based incontinence diagnoses in relation to menopausal status with a sufficient sample size. USI was the most common type of incontinence pre-menopause. We found a peculiar biphasic pattern of the age characteristics in women with pure stress incontinence (Fig. 1): USI peaked in the 46–50 age group, but there was a reduced prevalence from 50–61 years, as we hypothesised. However, there was an obvious increase in the 61–65 age group, before decreasing further with advancing age.

The age distribution of incontinence types were as expected. DO increased with age constantly until the age of 66–70 years, after which it decreased again. USI + DO increased steadily after menopause, eventually becoming the predominant type.

Similar to our study, several questionnaire-based studies have reported a reduction in SUI with increasing age [21, 22]. In order to understand why the biphasic pattern for USI in our study might occur, we note that the literature shows that peri-menopausal women tend to abandon vigorous physical exercise in order to protect against SUI. Dakic et al. recently showed that 41% of women with incontinence stop the exercise in which they had previously participated; furthermore, the older women were more likely to stop than the younger women (35% of women aged 18–25 years stopped exercise, 46% of those aged 26–45 years and 47% aged 46–65 years) [23]. It may be that women stop the activity that leads to their symptoms and only seek help when they experience additional symptoms of UUI, leading to the increase in USI with concomitant DO seen in our study. As is well published, DO rises with increasing age, which promotes a greater incidence of MUI in later life. Remission is another possible reason for the reduction in USI, or women may have had treatment for their condition. Women may also try to manage their symptoms by decreasing fluid intake and increasing voiding frequency [24].

Two Taiwanese studies [9, 10] have assessed age-related types of incontinence using urodynamics. Both included voiding dysfunction in their results and this was found to be a much higher rate (21% and 25.1% respectively) than for the general female population. The first study found that all types of UI have a biphasic peak at 41–50 and 51–60 years. The second study showed the highest prevalence of USI to be at the age of 40–49 years. They had very low rates of DO and USI + DO (7.3% and 4.5% respectively). The first study did not analyse menopause status. The second study mentioned that “menopause had no effect on UI”; however, no definition of menopause was given.

Of the publications that employed questionnaires, none of these large cross-sectional studies included data on menopause status. The EPINCONT surveyed 27,936 women evaluating different age groups and gave similar results to our study. SUI peaked at 45–49 years and remained the most predominant type until 59 years. MUI was highest in the over 60s (accounting for 40–48% of incontinence depending on age group). UUI increased with age, peaking at age 85–89 years, but the prevalence remained relatively low (7–23%) [21]. Hunskaar et al. [25] also used validated questionnaires in postal surveys and similarly found SUI to be the most prevalent type up to 59 years: 39% in the 18–44 group, 41% in the 45–59 group and 31% in ≥ 60s. MUI increased with age and was most common in > 60 years (41%). In contrast, in the EPIC study [4] SUI was the most prevalent type of UI in all age groups (≤ 39, 40–59 and ≥ 60 years).

Jahanlu and Hunskaar studied the natural history of UI in 2,229 middle-aged women aged 41–45 years at recruitment using questionnaires every second year over a 10-year period [24]. Age was grouped in 2-year intervals. UI peaked at 51–52 years and then a gradual decrease was seen in the 53–54 and 55+ groups. SUI was the predominant type until 55 years, which was different from our population (61–65). The reduction in incontinence was found to be due to a decrease in incidence as well as remission. The proportion of MUI increased with age and became the most common type in the last group (55+).

Mishra et al. studied a longitudinal cohort [22]. Questionnaires were posted annually from the age of 48 to 54 years; 1,525 women completed at least one questionnaire. Menopause status was assessed as follows: pre-menopause (still menstruating regularly), peri-menopause (3–12 months of amenorrhoea or irregular periods), and post-menopause (amenorrhoea for ≥ 12 months in the year preceding the questionnaire). The menopausal transition groups showed a higher percentage of SUI (pre-menopause to peri-menopause 52% and peri-menopause to peri-menopause 54%) compared with post-menopausal women (38%).

A number of studies have endeavoured to validate their questionnaires by performing urodynamics on patients who had completed questionnaires prior to the test. These have shown varying specificity and sensitivity [6, 26–28]. For example, Sandvik et al. [28] performed a study to validate their questionnaire diagnoses in relation to their urodynamics diagnoses. They investigated 250 incontinent women via questionnaire. Subsequently, bladder diaries were completed and a stress test, uroflow, and cystometry were performed. Sensitivity and specificity were calculated for the diagnoses of SUI, UUI, and MUI in women aged 20–50 and over the age of 50. Clinically (using urodynamics as well as clinical judgement), in the women aged 20–50 98 out of 148 (66%) had SUI, 12 out of 148 (8%) UUI and 28 out of 148 (19%) MUI compared with 29 out of 88 (33%), 29 out of 88 (33%) and 28 out of 88 (32%) in the over-50 group. Owing to the low specificity of the questionnaires, MUI was found to be overdiagnosed: a large proportion had genuine SUI when examined clinically.

Kirschner-Hermanns et al. found that short questionnaires frequently used in epidemiological studies had poor correlation with video urodynamics; SUI in particular was difficult to selectively isolate on the questionnaire [6].

Using completely different methods, a study in the USA employed their claims database for surgery to look at procedures for SUI in relation to age. They also found two peaks: the rates of SUI surgery were highest at the age of 46 and 70–71 [29]. Additionally, the age at prolapse surgery peaked at the same time as the second peak of SUI.

The present study is based on 2,994 urodynamics tests, which are the gold standard for diagnosing types of incontinence, rather than questionnaires, which are less precise. Sample size for the entire consecutive cohort of incontinent women having this test exceeded the required number. Another strength of this study is the comparison of the prevalence of type of incontinence in women living locally compared with those living outside of the area, which were similar, making our results widely generalisable. We were fortunate to have accurate data regarding systemic HRT use, which has been associated with a higher risk of stress incontinence [31]. Additionally, all practitioners involved in urodynamics testing were trained by the same urogynaecologist, and the tests were performed according to the ICS guidelines. Hence, the results of this study are derived from standardised methodology.

One of our limitations was a lack of precise recording of menopause onset (especially in hysterectomised women). However, the Appendix reveals the rates of the different types of incontinence using 51 years as the cut-off for menopause (Table 3) and excluding the 599 women with unclear menopause data (Table 4) with no significant differences when compared with the results in Table 2 using pre- and post-menopause where available and 51 years as the menopause cut-off when unknown. A further limitation is that our data reflect the urodynamics findings of 2,994 Australian women who underwent this test for incontinence, but may not reflect the actual prevalence of the conditions in relation to menopause in the population at large.

## Conclusion

In contrast to previous questionnaire-based studies, we have now shown that USI is the predominant type of leakage in pre-menopausal women, but the prevalence is significantly lower post-menopause. USI with concomitant DO increases after menopause, becoming the most common type of incontinence after the age of 66.

## Data Availability

The data that support the findings of this study are available from the corresponding author, [NtW], upon reasonable request.

## Appendix

**Table 3:** Type of urinary incontinence using 51 as cut-off age for menopause

Type of UI	< 51 years	≥51	Total	*p* value (Chi-squared)
USI	494 (59.0)	901 (41.8)	1,395	< 0.001
DO	131 (15.7)	393 (18.2)	524	< 0.001
USI + DO	212 (25.3)	863 (40.0)	1,075	< 0.001
Total	837	2,157	2,994	< 0.001

Data shown are *n* (%)

*UI* urinary incontinence, *USI* urodynamic stress incontinence, *DO* detrusor overactivity, *MUI* mixed urinary incontinence

**Table 4:** Type of urinary incontinence pre- and post-menopause excluding the 599 women with unclear data regarding menopausal status

Type of UI	Pre-menopause	Post-menopause	Total	*p* value (Chi-squared)
USI	455 (60.8)	730 (44.3)	1,185 (49.5)	< 0.001
DO	114 (15.3)	314 (19.1)	428 (17.8)	< 0.001
USI + DO	179 (23.9)	603 (36.6)	782 (32.7)	< 0.001
Total	748	1,647	2,395	< 0.001

Data shown are *n* (%)

*UI* urinary incontinence, *USI* urodynamic stress incontinence, *DO* detrusor overactivity, *MUI* mixed urinary incontinence
